# The ideal dairy cow for pasture-based systems

**DOI:** 10.3168/jdsc.2025-0910

**Published:** 2026-01-16

**Authors:** D.P. Berry

**Affiliations:** Teagasc, Animal & Grassland Research and Innovation Centre, Moorepark, Fermoy, Co. Cork, P61 P302, Ireland

## Abstract

Key characteristics of the ideal grazing dairy cow include (1) excellent reproductive efficiency and longevity, (2) a propensity for high voluntary DMI of grazed pasture which is efficiently converted to output, (3) the ability to achieve high output of valuable products (i.e., milk and meat) per unit area from grazed pasture, (4) excellent health, (5) easy care, (6) low environmental footprint, (7) being robust to environmentally induced perturbations without compromising output, and (8) producing progeny that can readily be finished early off pasture (i.e., achieve the desired carcass weight and fat cover with minimal supplementation). Genotype-by-environment interaction is a phenomenon where the traits' performance difference between 2 genotypes (i.e., individual animals, strains, breeds) differs between environments (i.e., pasture versus confinement dairy production systems). This phenomenon may manifest either as a reranking of genotypes for suitability (i.e., reranking) or as a change in the magnitude of their differences, with the most suitable genotype always excelling irrespective of the environment (i.e., rescaling). Although marked genotype reranking for individual traits is generally limited across environments, rescaling of the trait variances, combined with environment-specific differences in (1) which traits matter and (2) the relative importance of these traits, can lead to important reranking on overall dairy cow total merit indexes. Substantial exploitable genetic variation exists across a range of cow-level traits that determine suitability for pasture-based production systems. However, limited routine access to sufficient phenotypic data for certain traits continues to constrain progress toward delivering the ideal cow for such systems.

## Highlights

• Output per unit area is the critical performance measure, not output per cow.
• Pasture-based cows should have a high capacity to produce milk solids from pasture.
• Excellent reproductive performance is key to profitable pasture-based production.

## Graphical Abstract

The ideal cow for pasture-based systems should efficiently produce nutritious, high-value milk per hectare from grazed pasture, reproduce successfully within a seasonal system, be robust to external perturbations, and remain within the herd for many lactations, with minimal required intervention. The cow should be bred to suit the system, and not the system altered to suit the cow. Ample genetic variation is present in all animal-level characteristics, making it feasible to identify cows and their progeny that best fit individual production systems.

**Figure:**
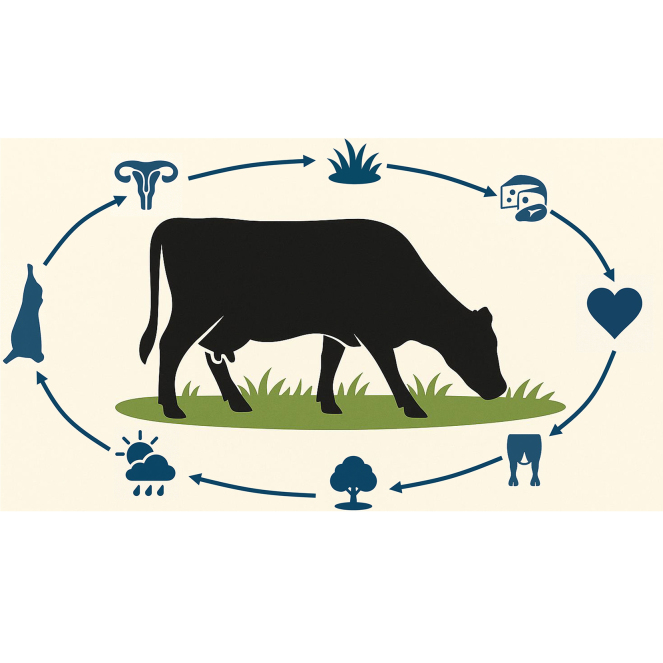

What is often referred to as cow type, a composite of genetic, physiological, and behavioral attributes, is a fundamental component of the efficient conversion of the available pasture resources into valuable output (i.e., milk and meat). The ideal cow (type) must achieve this within the main system constraints of the dynamic and less predictable nature of pasture quality and availability, along with weather and land quality, including other challenges such as walking distance across different terrains. Creating this ideal cow must be achieved in a production system that is a responsible environmental steward while also upholding the highest ethical standards. A mismatch between cow type and system demands often leads to an increased reliance on supplementation, veterinary intervention, and management input; these additional demands all erode the economic and sustainability competitive advantages of pasture-based dairy production.

This review outlines the key characteristics of the ideal dairy cow for pasture-based systems. Key characteristics of the ideal grazing dairy cow include (1) excellent reproductive efficiency and longevity, (2) a propensity for high voluntary DMI of grazed pasture which is efficiently converted to output, (3) the ability to achieve high output of valuable products (i.e., milk and meat) per unit area from grazed pasture, (4) excellent health, (5) easy care, (6) low environmental footprint, (7) being robust to environmentally induced perturbations, and (8) producing progeny that can readily be finished early off pasture (i.e., achieve the desired carcass weight and fat cover with minimal supplementation). Although these characteristics are relevant to both confinement-fed and pasture-based dairy cows, their relative importance varies between systems (Washburn and Mullen, 2014).

One of the defining features of pasture-based dairy production systems is the alignment between herd feed requirements and pasture growth and availability. Hence, the ideal dairy cow must calve within a narrow calendar window. Therefore, reproductive efficiency is a system-critical trait for most pasture-based production systems. Good productivity will not offset the failure to establish pregnancy within the designated breeding season; such cows are frequently culled. This is not the case in confinement dairy production systems, where calving tends to be year-round or split between 2 calving seasons in one year. To achieve compact calving, the cow must naturally return to estrus quickly after calving and then conceive, establish, and maintain pregnancy. (Short) gestation length is also a desirable characteristic to maintain a tight calving pattern. Sires with shorter gestation length can be used later in the breeding season so that late-bred cows will calve earlier and make the herd's calving period more compact. Six-week calving rate, which is the percentage of the herd that calves within the first 6 weeks of the herd's calving season, is a key performance indicator often used in pasture-based production systems; the target is 90%, ideally without any required interventions.

The performance of nulliparous heifers is frequently overlooked in dairy breeding goals. In seasonal-calving pasture-based systems, early onset of puberty to enable first calving at ~24 mo of age is essential to synchronize with the mature herd and exploit pasture availability. Albeit now dated, the recommended replacement rate for dairy herds was 18% (Esslemont and Peeler, 1993). Dividing 1 by 0.18 equates to the desired number of 5.56 lactations per cow. Assuming that 18% of the cow herd comprises first-parity cows, and 10% of those cows are culled before reaching second parity, with the culling rate per parity increasing by 2.3 percentage units per parity thereafter, then 6.7% of the herd would be expected to be parity 10 or greater. Therefore, reproductive efficiency and longevity are strongly intertwined in seasonal-calving pasture-based dairy herds. This has 2 implications. The first is that to achieve good longevity, good reproductive performance is a prerequisite. The second is that as reproductive performance improves, the importance of being able to maintain good production and health at older age becomes more important.

The low heritability of most farmer-recorded cow reproductive traits and longevity (Berry et al., 2014b) traditionally hindered genetic gain. However, as much genetic variability exits for reproductive traits as exists for milk production (Berry et al., 2014b). More extensive recording nowadays for reproductive traits, together with more widespread animal genotyping, has partly offset the influence of low heritability to achieving high accuracy of selection and, by extension, genetic gain for reproductive performance and longevity. Despite the generally higher heritability for the onset of puberty in dairy heifers (Lefebvre et al., 2021; Price et al., 2017), the difficulty in generating such data at scale limits the ability to achieve a high accuracy of selection and thus genetic gain (or indeed avoid genetic deterioration). Conveniently, genetic selection for improved cow fertility may, on average, also lower heifer age at puberty (Price et al., 2017), potentially reducing the need to directly phenotype puberty onset. However, recording of age at puberty could also be a useful early-left strategy to accelerate genetic gain in cow reproductive performance. Nonetheless, heifers that fail to calve early in the season (a composite of early puberty, estrus expression, conception, pregnancy establishment, and gestation length) are often culled, thereby functioning as a de facto selection pressure.

In conclusion, there are minimal technical barriers to delivering rapid genetic gain for reproductive performance in populations with a data recording system. Heterosis is typically most pronounced in reproductive traits, providing an additional and often rapid means of achieving superior phenotypic performance. Benefits from heterosis in a population do not necessarily require the maintenance of separate breeds, but can also be realized though the development of a composite breed; an example of this is the Kiwi Cross in New Zealand, which is a composite of a Holstein-Friesian and a Jersey.

When compared with indoor feeding systems, per cow nutrient intake in pasture-based systems tends to be restricted because high grazing intensity is maintained to preserve pasture quality and prevent excess pasture from entering the reproductive stage, which would reduce its nutritional value. Therefore, the ideal cow (and growing heifer) for grazing production systems must be able to consume large quantities of forage relative to its production potential; this will require animals that are motivated to graze. Although a high capacity to ingest grazed pasture is desirable, especially early in the season as milk production peaks, the ingested energy (and protein) must be converted to high-value output. Energy available to grazing dairy animals from what is ingested plus available from mobilized body tissue can be partitioned to milk production, growth (for heifers and first parity animals), activity, fetal growth, and general maintenance. General maintenance and activity are related to the (metabolic) weight of the animal. Hence, balancing intake capacity and maintenance requirements is critical. Accordingly, high voluntary pasture intake capacity does not necessarily equate to a high energy requirement. There is growing support for genetic selection on feed efficiency, reflecting the difference between actual feed intake and that required to maintain bodily functions. Mathematically, this description is equivalent to energy balance, which is the difference between actual energy intake and that required for bodily functions. The associations are well documented between negative energy balance, especially in early lactation, and reproductive performance and health (Veerkamp et al., 2000). Hence the impact on other traits from negative selection pressure on feed intake (not necessarily reduced feed intake) in pasture-based systems should be thoroughly explored before it is considered in a breeding program.

Considerable genetic variability exists in feed intake under pasture-based dairy cow production systems, even when adjusted for differences in milk production and body weight (Hurley et al., 2017). Unlike in confinement production systems, the accurate quantification of feed intake at pasture is not trivial. Heritability for feed intake in grazing dairy cows is relatively moderate (Hurley et al., 2017), but phenotypic intake data are still required to achieve a high accuracy of selection. Proxies for feed intake have been explored with varying success, such as the use of infrared data generated from milk samples (McParland and Berry, 2016) and animal-level sensors (Halachmi et al., 2016). Initiatives to collate dairy cow feed intake records across different populations have also been investigated (Berry et al., 2014a); the genetic correlations between feed intake measures in confinement-fed cows and grazing cows were notably weak (Berry et al., 2014a). In the absence of feed intake genetic evaluations, many countries include cow body weight or size with an associated negative weighting in their total merit index mainly to help deliver an efficient conversion of ingested feed to salable product. Although it is often assumed that a negative emphasis on cow size in many breeding objectives is selecting for smaller cows, this is not necessarily the case. Selection for high yields, which is ubiquitous in dairy cow breeding goals, will increase cow size; therefore, a negative emphasis on cow size in a total merit index may simply be mitigating this increase. Crucially though, the negative emphasis on cow live weight should not contribute to genetic selection for less body condition; including reproductive performance and health in the total merit index, or indeed body condition score itself (i.e., New Zealand Breeding Worth) may help to mitigate this risk, assuming selection is based on the total merit index.

Efficiency in pasture-based systems depends on balancing pasture availability per cow to meet her genetic potential while avoiding over-allocation, so as to minimize refusal rate and thereby maximize pasture utilization. Pasture-based production prioritizes hectare-level output from grazed pastures (with minimal supplementation) over per-cow yield. Given that hectare-level output is a function of individual cow output and stocking rate, breeding strategies differ from confinement systems, where the principal objective is output per lactation or per cow. In pasture-based production systems, moderate yields of high-quality milk per lactation are desirable, along with a salable calf. Because of the scarce pasture resources, partitioning proportionally more of the ingested energy into milk rather than body maintenance is important. Based on the mean performance metrics of a recent population of grazing Holstein-Friesian dairy cows in Ireland (Lahart et al., 2024), every 50-kg increase in cow live weight was expected to be associated with 1.58 kg DM greater intake; incidentally, this 50-kg heavier cow was also expected to eructate, on average, 28.5 g more enteric methane per day. Therefore, pasture-based dairy cows are small to moderately-sized, no doubt in part because feed intake (ability) as a proportion of live weight declines with increased cow size.

Milk in pasture-based systems (as in many countries) is paid for on a kilogram of fat plus protein basis (often simply referred to as milk solids). Many countries adopting pasture-based production systems are also dairy-exporting countries where less local demand exists for fluid milk, and thus fat and protein have higher market values. Except where sold as liquid milk, the product stream (e.g., cheese, butter, powders) is determined by fat and protein yield, not volume. Therefore, a higher milk solids concentration lowers costs both within the farm gate (e.g., storage, cooling) and outside the farm gate (e.g., processing, drying, hauling) per unit of saleable product. Because pasture typically has a lower energy density than concentrates, converting the ingested energy into saleable fat and protein is preferable to a greater liquid volume and lactose yield, which not only consume metabolic energy, but add limited value. Accordingly a high milk solids yield per unit area is critical. Moreover, fat and protein concentration is arguably even more important at pasture.

The existence of genetic variability in milk production in pasture-based dairy cows is well established, as is the genetic variability in milk solids output as a function of body weight (Berry and McCarthy, 2021), or indeed feed intake (Hurley et al., 2017). Genetic gain in milk production has been well documented across many dairy cow populations. This is aided by the large volumes of phenotypic data available for use in genetic evaluations coupled with the strong emphasis on milk traits within dairy cow total merit indexes (Cole and VanRaden, 2018).

Beef through the sale of surplus calves and cull cows contributes to the profit margin of dairy farms. Using simple calculations, Berry (2021) concluded that the contribution of beef to the gross output per unit of milk is, on average, greater in lower-yielding herds and when beef price is high. Given the generally lower yield in pasture-based herds, the contribution of beef to the gross output per unit milk solids may be expected to be greater. The popularity of beef-on-dairy matings is intensifying, and the capacity to mate proportionally more dairy females to beef sires is greater when herd reproductive performance is high, as in many pasture-based herds. The dam, however, still contributes half the genetic potential to her beef calves, and an unfavorable genetic trend in beef traits has been reported in some pasture-based dairy herds (Mulhall et al., 2023). This can be offset if beef sires make equally rapid genetic progress in the favorable direction, or if a higher proportion of beef-on-dairy matings are used. Considerable interbreed and intrabreed differences in beef merit exist among beef sires used for beef-on-dairy matings (Berry and Ring, 2020). There is less scope to offset the genetic trend toward lower cull cow carcass output, but again, if reproductive performance is high, then the rate of culling will be lower. Nonetheless, genetic variance in cull cow carcass traits, including dressing percentage, is known to exist in pasture-based cows (Coyne et al., 2019).

Despite commonality in health conditions between grazing and confinement systems, some health conditions are system-specific or occur at higher frequency in pasture-managed cows. Climate change may also increase the (future) risk of new or exotic conditions for animals kept outdoors. Health conditions more relevant to grazing animals include hypomagnesemia, bloat, facial eczema, photosensitization and tuberculosis as well as exposure to certain endo and ectoparasites. Because pasture nutrient content is inherently variable, micronutrient imbalances (i.e., deficiencies or excesses) and related disorders may be more frequent in pasture-fed cows, reflecting the difficulty of precisely balancing the micronutrient content of the diet. Even for general health ailments common between pasture-based and confinement production systems, nuances of the ailment may differ; lameness is a good example, in that grazing cows walk long distances on softer soils, whereas confined cows spend extended periods standing on artificial surfaces. Low morbidity and mortality are also important in young animals, not just for maximizing profit, but also for meeting the ethical and societal expectations of responsible farming.

Although it is mostly based on confinement dairy production systems, the heritability for health traits generally tends to be low (Koeck et al., 2012; Berry et al., 2011). Improving the accuracy of recording, including improvements to the sensitivity and specificity of the recorded data, should also be prioritized as a means of increasing the heritability, thereby contributing to a higher accuracy of selection, even if the extent of data recording is poor (Bishop and Woolliams, 2010). Accordingly, health trait recording should be prioritized in order to deliver a high accuracy of selection. Once high accuracy is secured, the magnitude of the additive genetic variance becomes the principal determinant of potential genetic gain. Therefore, the main factor affecting the phenotypic and, by extension, genetic variance of health traits in pasture-based production systems, is the incidence of the conditions. The phenotypic variance of a binary trait $\left(\sigma_{p}^{2}\right)$ is

$$\sigma_{p}^{2}=p\left(1-p\right),$$

where *p* is the incidence of the disease. Therefore, assuming an incidence of 5% with a heritability of 3%, the genetic SD of the condition on the liability scale is expected to be 0.038. This equates to a mean incidence of 4.45% and 5.56% in the genetically best 20% and worst 20%, respectively. Hence, exploitable additive genetic variability clearly exists, which could be complemented by leveraging nonadditive genetic effects, especially through crossbreeding. Heterosis effects can be quite substantial for health traits in dairy cows (Sørensen et al., 2008), especially if one of the breeds used has been selected for greater health (i.e., breeds or animals originating from Scandinavian countries).

Grazing dairy cows spend most if not all of the year outdoors, making continuous observation inherently more difficult, as well as leading to greater exposure to weather. Therefore, ease of care is essential in pasture-based systems. Remote monitoring technologies offer solutions (albeit at cost), and although early adoption was constrained by connectivity and power limitations, these constraints are diminishing. Around parturition, minimal dystocia and excellent neonatal vitality and viability are essential. Additional easy care attributes include polledness, docility, milking duration, and maternal behavior. Polledness is controlled by genetic variants (Schafberg and Swalve, 2015), with large genetic variability also known to exist in pasture-based dairy cow populations for dystocia (Berry and Ring, 2020) and docility (Berry et al., 2013). Berry et al. (2013) documented a genetic SD for milking duration in grazing dairy cows independent of milk yield of 36.8 s; this accumulates to 62 h over a 305-d lactation for cows milked twice daily in a herringbone parlor with 10 rows per milking. Furthermore, colostrum quality, principally its immunoglobulin concentration, is critical to calf survival and reduces the need for human intervention, provided newborns receive timely access to sufficient high-quality colostrum. Heritable genetic variation in immunoglobulin concentration in pasture-based dairy cows has been documented (Conneely et al., 2013).

Functional locomotion and mammary conformation are central to easy care management, with the optimal conformation profile varying subtly between grazing and confinement herds. Exploitable genetic variability in cow conformation among pasture-based dairy cows has already been documented (Cue et al., 1996) with many populations routinely assessing cow conformation. Grazing dairy cows in Ireland have to walk almost 2 km daily just between the field and the milking parlor; over a 305-d lactation, this equates to an annual walk (not including grazing in the paddock) of over 600 km, or a total of almost 2,500 km over 4 lactations (ignoring heifers and dry cows); the terrain can be uneven, of different gradients, and can change from being soft to hard underfoot. It is expected that conformation will gain importance as cows age, and therefore be of greater importance in production systems with good cow longevity. Using a population of 52,477 pasture-based Irish dairy cows, Williams et al. (2022) reported that the genetic correlation between survival to the next lactation and a selection of 3 mammary conformation traits strengthened with parity. A similar strengthening of genetic correlations with conformation as cows age was documented in New Zealand pasture-based cows (Stachowicz et al., 2017)

The main sources of environmental pressure from grazing dairy production systems are methane (enteric and from manure), nitrogen leaching, and water usage, as well as the effects on land compaction and biodiversity. Although considerable genetic variability in enteric methane emissions has been demonstrated to exist in (confinement-fed) dairy cows (Pszczola et al., 2017), it is the extent of genetic variability independent of traits already under direct selection that is important to quantify the achievable response to direct selection. Moreso, the genetics of enteric methane emissions in dairy cows have exclusively focused on daily emissions, although lifetime emissions, and arguably per unit of lifetime output, are most important. Measuring methane emissions, nitrogen leaching, and even water intake is complex in all systems, but especially so in pasture-based systems. Therefore, the main barrier to genetic improvement is access to reliable, individual-animal phenotypes for these traits. Nonetheless, the technologies and standard operating procedures for measuring these traits do exist.

Animals should be robust to environmentally induced indirect and direct perturbations. This is because pasture-based production systems tend to be more restrictive, less stable, and subject to greater unpredictable disturbances than indoor production systems. Rising temperatures, changes in patterns of precipitation, and extreme weather events are likely to affect grazing dairy cows more through both direct effects (e.g., heat stress, parasite burden) and indirect effects (i.e., both the quality and quantity of the forage available). Dairy cows, therefore, need to be more robust and resilient to such perturbations. Robustness can be defined as the ability of an animal, when faced with environmental challenges, to carry on doing what it needs to do to favor its future ability to reproduce (Friggens et al., 2017). In the face of external perturbations, the dairy cow must initiate adaptive responses, which could include a change in performance, physiology (e.g., nutrient allocation), or behavior. A temporary reduction in feed availability is a common annual occurrence in pasture-based production systems, so the cow must be able to endure this period of feed deficit and recover quickly and fully once conditions become favorable. Supplementation is often used as a short-term strategy to compensate for feed deficits; therefore, the cow must have the ability to maintain performance during this diet transition. Strategies and the consequences of genetic selection for resilience in dairy cows has been explored (Bengtsson et al., 2022). Body condition score is an indicator of the animal's body energy reserves, which can be mobilized to offset periods of negative energy balance; body condition score in grazing dairy cows has been documented to be heritable (Pryce and Harris, 2006). Genetic evaluations for heat tolerance exist in some countries (e.g., Nguyen et al., 2017), acting as a useful tool to identify germplasm that can maintain productivity even when the temperature-humidity index is high; this is especially true given that unfavorable genetic trends have existed in some countries (Nguyen et al., 2017). Heat tolerance is particularly important in pasture-based systems because control of the environment through misting systems and fans is not possible. Smaller cows potentially have an advantage here because of their higher ratio of surface area to volume.

Many beef abattoirs impose carcass specifications on criteria such a weight, conformation, and fat. These are imposed to ensure the processing cost per carcass is diluted across enough saleable meat yield (i.e., minimum carcass weight) and that the primal meat cuts are appealing to the consumer. Success, therefore, depends on meeting these carcass specifications predominantly from forage with minimal supplementation; performance, like with the dairy system, is measured on a per-unit-area basis and not per animal. Earlier off-pasture finishing reduces housing days and, with that, the requirements for slurry storage, mechanical feeding, fuel, and other associated costs. Cattle that reach market specification (i.e., minimal carcass weight and fat cover) early at pasture must combine high voluntary pasture intake with efficient conversion to carcass gain. Ideally, these animals should mature early to ensure a sufficient fat cover on the carcass for delivering on consumer expectations with minimal supplementation. Although the use of beef-on-dairy is increasing, half the genes in these calves originate from the (dairy) dam. Genetic variability in maturation rate in growing dairy heifers in pasture-based systems is known to exist (Berry et al., 2005), as are differences in carcass characteristics (Berry et al., 2021).

Although genetic variability exists in dairy cow characteristics pertinent to pasture-based systems, the magnitude of these differences across breeds, strains, or individual animals, as well as their respective relative rankings, can vary depending on the production environment. Genotype-by-environment interactions may be defined as the phenomenon that performances of different genotypes are not equally affected by different environments (Falconer, 1952). Genotype-by-environment interactions can materialize as a scaling effect, where the relative size of the difference among individuals changes across environments, or as a reranking effect, where the ranking of individuals for the same trait shifts because the genetic correlation between environments is less than one. The greater the genetic divergence among individuals or the larger the differences between the environments (i.e., grazing versus confinement) being compared, the greater the likelihood of genotype-by-environment interactions existing (Falconer, 1989). Although the extent of detected genotype-by-environment interactions is currently relatively small for many individual dairy traits (Craig et al., 2018), the acceleration in genetic gain in some breeds for milk production, but also the ever-improving cow diet formulation in indoors production system, could lead to genotype-by-environment interactions becoming more pronounced in the future with reranking of animals.

Quantifying the type and extent of genotype-by-environment interactions is important because it may affect genetic gain. If only a rescaling effect exists, then the best individuals for a given trait remain the same across environments, and just the magnitude of genetic gain will differ by environment. However, if the genotype-by-environment interaction is due to reranking and is not considered in the breeding program, then genetic gain from using germplasm from the other environment will be reduced, and in fact could actually be unfavorable if the genetic correlation between the selection criteria in the 2 environments is negative. Interbull estimates the genetic correlations between a series of dairy production traits in different countries. The estimated genetic correlations in Holsteins among either milk yield, fat yield, or protein yield between 31 countries varies from 0.75 to 0.97 (Interbull, 2025a), whereas the genetic correlations between fertility traits in 19 different countries are as weak as 0.24, with an average of 0.75 (Interbull, 2025b). Hence, the extent of reranking of animals across countries is expected to be greater for reproductive traits than for production traits.

Scaling effects observed for individual traits may ultimately result in a reranking of dairy animals when evaluated on a composite breeding index that incorporates those traits. Reranking of animals on breeding objectives from different countries or production systems can be due to (1) different component traits or even definitions of the traits in the respective indexes, including different statistical models used in genetic evaluations; (2) different relative emphasis on traits within the respective breeding objectives; (3) different genetic variances for a given trait between environments; or (4) reranking genotype-by-environment interactions for individual traits. The expected correlation (r) between 2 total merit indexes where the trait (co)variances are different can be estimated as

$$r=\frac{\omega^{'}\cdot D\cdot Covar\cdot \omega }{\sqrt{\left(\omega^{'}\cdot Covar\cdot \omega \right)\left(\omega^{'}\cdot D\cdot Covar\cdot D\cdot \omega \right)}},$$

where **ω** is the vector of trait weights; **D** is a diagonal matrix with elements of the square root of t_i_, with t_i_ being the variance rescaling factor for trait i; and **Covar** is the genetic covariance matrix among the traits. Assuming a 3-trait index where each trait has the same variance (i.e., 1) and the correlations between all traits are 0.1; the weight on the first, second, and third trait is 1, 1 and 3, respectively. If the variance for traits 2 and 3 is 0.5 and 0.1 times that of the first index, then the correlation between both indexes simply due to a change in (co)variances is 0.91. The correlation between 2 total merit indexes differing in the weightings per trait within the index, or indeed the actual traits in the index (i.e., a weight of zero used for the trait(s) in question), can be calculated as

$$r=\frac{{\omega^{'}}_{a}\cdot Covar\cdot \omega_{b}}{\sqrt{\left({\omega^{'}}_{a}\cdot Covar\cdot \omega_{a}\right)\left({\omega^{'}}_{b}\cdot Covar\cdot \omega_{b}\right)}},$$

where **ω***_a_* and **ω***_b_* are the vectors of weights in index a and index b, respectively. Assuming again a 3-trait index with equal weights, each with a variance of 1, and a covariance of 0.1 between each pair of traits, if the weight on one of the 3 traits is 5 times greater (e.g., fertility in pasture-based herds), then the correlation between the 2 indexes is expected to be 0.82. If, in fact, this trait was not actually in the other index, then the correlation between both indexes weakens to 0.40.

September 2025 genetic evaluations for individual Holstein-Friesian sires born since the year 2000 were retrieved for the United States, Canada, Australia, Ireland, the United Kingdom, the Netherlands, and New Zealand. The partial correlation between pairs of indexes was calculated for animals with a reliability >70% in the pair of countries; the correlation was adjusted for genetic trends through the inclusion of year of birth of the bull as a class fixed effect. The correlations among the indexes are shown in Table 1. The mean correlation between the Total Performance Index (**TPI**; United States), Net Merit Index (**NMI**; United States), Lifetime Profit Index (**LPI**; Canada), Dutch-Flemish Index (**NVI**; the Netherlands and Flanders), and Profit Lifetime Index (**PLI**; United Kingdom) was 0.74; the mean correlation among the 4 grazing-based indexes was 0.65. The mean correlation between the grazing indexes with the TPI, NMI, LPI, NVI and PLI was 0.61; the mean correlation between the latter group of indexes with both the Economic Breeding Index (**EBI**; Ireland) and Breeding Worth (**BW**; New Zealand), both of which are based on genetic and economic models representing of grazing dairy herds, was 0.49. Hence, there is expected to be considerable reranking of Holstein-Friesian bulls ranked on breeding indexes typical of confinement dairy production systems versus those representing pasture-based production systems. Some of the indexes were, in fact, poorly correlated (e.g., 0.39 between the BW of New Zealand and the PLI of the United Kingdom), likely due to rescaling of genetic variances for each trait, differences in what traits comprised the respective indexes, differences in the definitions of traits (e.g., fertility traits), and differences in the relative emphasis on each trait included in the respective index.

**Table 1:** Correlations (lower diagonal) and number of Holstein-Friesian artificial insemination sires (upper diagonal) contributing to that correlation between the total merit dairy indexes^1^ of various countries

Index	TPI	NMI	LPI	NVI	PLI	BPI	GMI	EBI	BW	SCI
TPI		36,099	7,257	2,062	25,886	4,015	36,099	3,173	1,184	25,886
NMI	0.87		7,009	1,770	25,094	3,475	36,716	2,908	1,130	25,094
LPI	0.73	0.61		817	7,183	1,697	7,009	1,205	566	7,183
NVI	0.73	0.71	0.69		2,236	1,022	1,770	1,233	520	2,236
PLI	0.75	0.75	0.73	0.81		4,057	25,094	3,498	1,418	27,144
BPI	0.58	0.51	0.64	0.57	0.71		3,475	2,432	1,216	4,057
GMI	0.85	0.99	0.59	0.70	0.76	0.52		2,908	1,130	25,094
EBI	0.43	0.47	0.50	0.51	0.61	0.56	0.53		1,050	3,498
BW	0.46	0.57	0.43	0.45	0.43	0.51	0.58	0.62		1,418
SCI	0.58	0.62	0.59	0.66	0.90	0.66	0.67	0.83	0.63	

1 TPI = Total Performance Index (United States); NMI = Net Merit Index (United States); LPI = Lifetime Profit Index (Canada); NVI = Dutch-Flemish Index (the Netherlands and Flanders); PLI = Profit Lifetime Index (United Kingdom); BPI = Balanced Performance Index (Australia); GMI = Grass Merit Index (United States); EBI = Economic Breeding Index (Ireland); BW = Breeding Worth (New Zealand); SCI = Spring-Calving Index (United Kingdom).

Within country, the correlation between the national US dairy breeding index (i.e., the NMI) and US grazing breeding index was 0.99; differences in the weighting factors per trait are the only difference between these indexes (Council on Dairy Cattle Breeding, 2025). The correlation between the national United Kingdom dairy breeding index (the PLI) and the United Kingdom grazing breeding index (the Spring-Calving Index) was 0.90; as well as different economic values per trait in both indexes, the predicted transmitting ability estimates from the national genetic evaluations for milk production in the United Kingdom are rescaled to have a lower variance for use in the Spring-Calving Index.

Selective breeding is a proven, evidence-based approach for altering cow type. However, genetic change is not synonymous with genetic progress; the latter only occurs when the breeding goal is well specified and aligned with the production system. The pillars of a successful breeding program include: (1) a clearly defined breeding objective; (2) robust, validated genetic evaluations for the traits in the selection index; (3) an efficient and effective breeding scheme; and (4) rapid dissemination of elite germplasm into commercial herds supported by clear and value-adding decision supporting tools. For pasture-based systems, formulating breeding goals is as complex as in confinement systems, yet entirely tractable with the well-established methodology. However, the guiding principle in breeding pasture-based dairy cows is that the system should define the ideal cow, not the other way around. An appropriate and futuristic breeding goal from which to guide selection decisions is therefore key.

Genetic evaluations—but more importantly, accurate genetic evaluations—are a function of the quantity (and structure) of information available and the heritability of the trait(s) of interest; information here includes data on the phenotype or genotype itself, but also the nuisance factors that contribute to the nongenetic variability in the trait. Information could also include data on correlated traits with a known covariance structure with the trait(s) of interest. The interplay between heritability and available information is a major contributor to the speed at which breeding can deliver the ideal cow. Traits where achieving rapid genetic gain is challenging generally fall into 2 categories: (1) low-heritability traits with poorly recorded data, and (2) moderate heritability traits with accurate, less frequent recording. In populations where data come entirely from pasture systems, each record is fully informative; however, when data originate predominantly from indoor systems, informativeness scales with the genetic correlation to pasture conditions (i.e., the extent of reranking). Given the challenges of recording some phenotypes under grazing production systems, there may be a greater need for specialized and well-designed phenotyping herds for grazing systems, especially for the difficult to measure traits. If well-constructed, these herds could also serve as sources of the next generation of germplasm for the national breeding program.

Although exceptions exist, health conditions recorded by farmers (e.g., mastitis, lameness) generally tend to be lowly heritable and are not well recorded in many populations; this makes them an example of a trait with both low heritability and poor data quality. Contributing factors to the low heritability include misdiagnosis, vague trait definitions, or under recording by producers. In contrast, more objective measures of animal health such as antibody mediated responses or ELISA-based results tend to be more heritable and, if data are routinely collected, this information could be included in national multitrait genetic evaluations for animal health (e.g., somatic cell count as a predictor of mastitis). Capturing veterinarian diagnoses of conditions for use in genetic evaluations should be one of the highest priorities for animal breeding because heritability estimates based on veterinary diagnosed conditions are expected to be higher because they are more accurately recorded. However, in low-cost, pasture-based systems, producers may not consistently seek veterinary input, leading to continued under-reporting.

Examples of moderately heritable traits that are costly to measure include age at puberty, feed intake, nitrogen use efficiency, and methane emissions. Although lower-cost proxy measures merit exploration, the cost of measurement on a subpopulation of animals should be considered within the context of the potential cumulative and permanent benefit to the entire population. Although proxies for such traits in grazing dairy cows have been proposed (McParland and Berry, 2016), the opportunity cost of lost genetic gain from direct selection on the actual traits of interest should be quantified. A good example is the former use of cow linear type traits as genetic predictors of cow size or expected longevity; the usefulness of these traits has been superseded by the actual phenotypes themselves. In the dairy sector, the cost of phenotyping, most of which could be incurred upfront, could run into the millions, but this pales in significance when the industry is worth billions. When considered on a per-liter of milk or per-kilogram of milk solids basis, this investment is also often negligible. If no substantial return on investment can be demonstrated, then the value of breeding for such traits must be questioned. There is a strong rationale for public or public–private partnerships to provide the initial funding for research that establish the extent of genetic variability, develop standard operating procedures, and quantify phenotyping costs. Once the business case is established, ongoing deployment should be funded by the beneficiaries. Given the proven track record of genetic improvement in driving year-on-year gains in performance, such justification should be straightforward. Moreover, because producers already directly pay for (or automatically generate) data on most traits included in dairy total merit indexes, modest additional investment in phenotyping, data collation, and associated infrastructure by breeding bodies appears to be an entirely reasonable request.

Large candidate populations are essential for achieving high selection intensity and, consequently, faster genetic gain. In predominantly pasture-based countries such as Ireland and New Zealand, the presence of large dairy populations provides a substantial pool from which to identify elite germplasm. In these settings, there is often a single or predominant breeding objective, enabling all or most of the resources to be concentrated on achieving gain in just the pasture-based total merit index. In contrast, countries or client bases dominated by indoor production systems may face competing breeding objectives albeit these are often correlated. If resources are limited, genetic progress for the majority of producers may be compromised by efforts to support a pasture-based breeding program. In such cases, faster progress may actually be achieved by strategically sourcing genetically elite bulls (and potentially embryos) from populations focused on pasture-based dairy production. Ultimately, the average rate of genetic gain achievable will depend on both the genetic correlation between the breeding objectives across countries and the rate of genetic gain being realized in the donor population.

In summary, cow type is not a passive input, but a dynamic system component. It influences not only direct production metrics like milk yield and reproductive success, but also the broader efficiency, resilience, and sustainability of the farm system. Selecting or breeding for an appropriate cow type is therefore central to optimizing outcomes in grazing-based production environments.

## References

[bib1] Bengtsson C., Thomasen J.R., Kargo M., Bouquet A., Slagboom M. (2022). Emphasis on resilience in dairy cattle breeding: Possibilities and consequences. J. Dairy Sci..

[bib2] Berry D.P. (2021). Invited review: Beef-on-dairy—The generation of crossbred beef× dairy cattle. J. Dairy Sci..

[bib3] Berry D.P., Bermingham M.L., Good M., More S.J. (2011). Genetics of animal health and disease in cattle. Ir. Vet. J..

[bib4] Berry D.P., Coffey M.P., Pryce J.E., De Haas Y., Løvendahl P., Krattenmacher N., Crowley J.J., Wang Z., Spurlock D., Weigel K., Macdonald K., Veerkamp R.F. (2014). International genetic evaluations for feed intake in dairy cattle through the collation of data from multiple sources. J. Dairy Sci..

[bib5] Berry D.P., Coyne J., Coughlan B., Burke M., McCarthy J., Enright B., Cromie A.R., McParland S. (2013). Genetics of milking characteristics in dairy cows. Animal.

[bib6] Berry D.P., Evans R.D., Kelleher M.M. (2021). Prediction of genetic merit for live weight and body condition score in dairy cows using routinely available linear type and carcass data. J. Dairy Sci..

[bib7] Berry D.P., Horan B., Dillon P. (2005). Comparison of growth curves of three strains of female dairy cattle. Anim. Sci..

[bib8] Berry D.P., McCarthy J. (2021). Contribution of genetic variability to phenotypic differences in on-farm efficiency metrics of dairy cows based on body weight and milk solids yield. J. Dairy Sci..

[bib9] Berry D.P., Ring S.C. (2020). Observed progeny performance validates the benefit of mating genetically elite beef sires to dairy females. J. Dairy Sci..

[bib10] Berry D.P., Wall E., Pryce J.E. (2014). Genetics and genomics of reproductive performance in dairy and beef cattle. Animal.

[bib11] Bishop S.C., Woolliams J.A. (2010). On the genetic interpretation of disease data. PLoS One.

[bib12] Cole J.B., VanRaden P.M. (2018). Possibilities in an age of genomics: The future of selection indices. J. Dairy Sci..

[bib13] Conneely M., Berry D., Sayers R., Murphy J., Lorenz I., Doherty M., Kennedy E. (2013). Factors associated with the concentration of immunoglobulin G in the colostrum of dairy cows. Animal.

[bib14] Council on Dairy Cattle Breeding (2025). Net merit as a measure of lifetime profit: 2025 revision. https://uscdcb.com/wp-content/uploads/2025/02/nmcalc-2025ARR-NM9without-typecomposites.pdf.

[bib15] Coyne J.M., Evans R.D., Berry D.P. (2019). Dressing percentage and the differential between live weight and carcass weight in cattle are influenced by both genetic and non-genetic factors. J. Anim. Sci..

[bib16] Craig H.J.B., Stachowicz K., Black M., Parry M., Burke C.R., Meier S., Amer P.R. (2018). Genotype by environment interactions in fertility traits in New Zealand dairy cows. J. Dairy Sci..

[bib17] Cue R.I., Harris B.L., Rendel J.M. (1996). Genetic parameters for traits other than production in purebred and crossbred New Zealand dairy cattle. Livest. Prod. Sci..

[bib18] Esslemont R.J., Peeler E.J. (1993). The scope for raising margins in dairy herds by improving fertility and health. Br. Vet. J..

[bib19] Falconer D.S. (1952). The problem of environment and selection. Am. Nat..

[bib20] Falconer D.S. (1989).

[bib21] Friggens N.C., Blanc F., Berry D.P., Puillet L. (2017). Review: Deciphering animal robustness. A synthesis to facilitate its use in livestock breeding and management. Animal.

[bib22] Halachmi I., Ben Meir Y., Miron J., Maltz E. (2016). Feeding behavior improves prediction of dairy cow voluntary feed intake but cannot serve as the sole indicator. Animal.

[bib23] Hurley A.M., López-Villalobos N., McParland S., Lewis E., Kennedy E., O'Donovan M., Burke J.L., Berry D.P. (2017). Genetics of alternative definitions of feed efficiency in grazing lactating dairy cows. J. Dairy Sci..

[bib24] Interbull (2025). APPENDIX I. Sire standard deviations in diagonal and genetic correlations below diagonal. https://interbull.org/static/mace_evaluations_archive/eval/prod-appen1-141.pdf.

[bib25] Interbull (2025). APPENDIX I. Sire standard deviation (diagonal) and genetic correlations (below diagonal) considered in December 2011 Routine Evaluation. https://interbull.org/static/mace_evaluations_archive/Female_fert/fert-appen1-113.htm.

[bib26] Koeck A., Miglior F., Kelton D.F., Schenkel F.S. (2012). Health recording in Canadian Holsteins: Data and genetic parameters. J. Dairy Sci..

[bib27] Lahart B., Buckley F., Herron J., Fitzgerald R., Fitzpatrick E., Galvin N., Shalloo L. (2024). Evaluating enteric methane emissions within a herd of genetically divergent grazing dairy cows. J. Dairy Sci..

[bib28] Lefebvre R., Larroque H., Barbey S., Gallard Y., Colleau J.J., Lainé A.L., Boichard D., Martin P. (2021). Genome-wide association study for age at puberty and resumption of cyclicity in a crossbred dairy cattle population. J. Dairy Sci..

[bib29] McParland S., Berry D.P. (2016). The potential of Fourier transform infrared spectroscopy of milk samples to predict energy intake and efficiency in dairy cows. J. Dairy Sci..

[bib30] Mulhall S.A., Sleator R.D., Evans R.D., Twomey A.J. (2023). Genetic and phenotypic trends for carcass traits in Irish beef cattle. Ir. J. Agric. Food Res..

[bib31] Nguyen T.T.T., Bowman P.J., Haile-Mariam M., Nieuwhof G.J., Hayes B.J., Pryce J.E. (2017). Short communication: Implementation of a breeding value for heat tolerance in Australian dairy cattle. J. Dairy Sci..

[bib32] Price M.D., Camara M.D., Bryant J.R., Meier S., Burke C.R. (2017). Genetic parameters of puberty estimated using two genetically divergent groups of Holstein-Friesian dairy heifers. Proc. Assoc. Advmt. Anim. Breed. Genet..

[bib33] Pryce J.E., Harris B.L. (2006). Genetics of body condition score in New Zealand dairy cows. J. Dairy Sci..

[bib34] Pszczola M., Rzewuska K., Mucha S., Strabel T. (2017). Heritability of methane emissions from dairy cows over a lactation measured on commercial farms. J. Anim. Sci..

[bib35] Schafberg R., Swalve H.H. (2015). The history of breeding for polled cattle. Livest. Sci..

[bib36] Sørensen M.K., Norberg E., Pedersen J., Christensen L.G. (2008). Invited Review: Crossbreeding in dairy cattle—A Danish perspective. J. Dairy Sci..

[bib37] Stachowicz K., Quinton C., Meyer S., Amer P.R., Phyn C.V.C. (2017). Genetic parameters of cow functional survival and correlations with predictor conformation and farmer-opinion traits with recommendations for genetic evaluation. Interbull Bull..

[bib38] Veerkamp R.F., Oldenbroek J.K., Van der Gaast H.J., Van der Werf J.H.J. (2000). Genetic correlations between days until start of luteal activity and milk yield, energy balance, and live weights. J. Dairy Sci..

[bib39] Washburn S.P., Mullen K.A.E. (2014). Invited review: Genetic considerations for various pasture-based dairy systems. J. Dairy Sci..

[bib40] Williams M., Sleator R.D., Murphy C.P., McCarthy J., Berry D.P. (2022). Re-assessing the importance of linear type traits in predicting genetic merit for survival in an aging Holstein-Friesian dairy cow population. J. Dairy Sci..

